# Synthesis and Characterization of Schiff Base Polymers via Metal Coordination and Its Application in Infrared Stealth Coating

**DOI:** 10.3390/polym14214563

**Published:** 2022-10-27

**Authors:** Xiangyu Li, Lishuai Zong, Weijie Li, Yibo Wang, Jinyan Wang, Xigao Jian

**Affiliations:** State Key Laboratory of Fine Chemicals, Liaoning High Performance Resin Engineering Research Center, Department of Polymer Science & Engineering, Dalian University of Technology, Dalian 116024, China

**Keywords:** Schiff base, coordination polymer, coating, infrared emissivity, stealth

## Abstract

In order to reduce the infrared emissivity to meet the requirements of modern warfare for infrared stealth materials, we prepared the polymers containing Schiff base moieties using polyetheramine and 2,6-pyridinedicarboxaldehyde by solution polycondensation and coordinated with Ni^2+^, Cu^2+^, and Sm^3+^ ions to prepare organic coatings. The structure and the thermal and mechanical properties of the coatings were studied in detail. Meanwhile, the effect of the conductivity change of coordination polymers on infrared emissivity was studied systematically. The results showed the polymer coordinated with Sm^3+^ ions had the lowest energy band gap, which was 2.99 eV, and the best electrical conductivity of 3.54 × 10^−4^ S/cm compared with Ni^2+^ and Cu^2+^ coordination polymers. The infrared emissivity was the lowest in the 2–22 μm infrared waveband range, which reached 0.58, suggesting the polymers containing Schiff base moieties and their coordination polymers may have a great potential to be applied as infrared stealth materials in military applications.

## 1. Introduction

With the advancement of science and technology, infrared detection technology has become increasingly sophisticated. In particular, the advent of infrared detection and remote sensing equipment, with high detection accuracy and high resolution, has caused huge threats to military facilities and weapons [1,2,3]. To minimize the difference between the target and the background, it’s necessary to preserve the infrared stealth technology of military targets [4,5]. According to the Stefan–Boltzmann law, the intensity of the mid-far infrared signal emitted from an object is proportional to the surface emissivity (ε) and the fourth power of absolute temperature (T). Therefore, controlling temperature and surface emissivity are two means to achieve mid-far infrared stealth [6,7]. However, temperature control is too difficult because this method requires additional cooling and heating equipment and increases the weight of the aircraft [8,9]. In contrast, controlling the surface emissivity is a relatively simpler method, which can be achieved through coating infrared stealth materials on objects to effectively suppress mid-far infrared signals [10,11]. Nowadays, infrared stealth materials, such as inorganic or metallic materials, are widely used because of their high reflectivity in the infrared band [12,13]. Wang et al. [14] prepared a core-shell structural SiO2@DNA-LDH nanocomposite via layer-by-layer assembly and it exhibited a much lower infrared emissivity, as low as 0.458 at a wavelength of 8–14 μm. Qin et al. [15] obtained infrared laser-compatible stealth materials by coating aluminum powder with antimony-doped tin oxide (ATO), whose emissivity was 0.708 at 8–14 μm. However, there are some disadvantages to these materials, such as high processing costs, complicated preparation processes, and poor stability [16,17].

In order to meet the requirements of modern warfare for lightweight, low-cost processing, and multi-spectral response, stealth materials are exploited. Organic polymer materials have evoked wide interest in recent years due to their low density, good ductility, and tunable optical properties [18,19,20]. The infrared emissivity can be changed to realize the possibility of various applications by adjusting the structure and composition of organic polymers [21,22]. Pan et al. [23] prepared optically active poly(N-propargylamide)s bearing stigmasterol moieties by varying the feed ratio of chiral and achiral monomers, and the emissivity ranged from 0.536 to 0.798 at a wavelength of 8–14 μm. Louet et al. [24] investigated a series of poly(3,4-ethylene-dioxythiophene) with different morphologies and conductivities, whose infrared reflectance could be tuned from 21 to 67% in the range of 8–20 μm.

According to the Hagen–Rubens approximation theory at a low frequency and Kirchhoff’s law, it is noted that materials with higher electrical conductivity may have lower infrared emissivity [25,26]. Among them, Schiff base materials have excellent coordination capacity due to the lone pair electrons of N atoms in the hybrid orbital that can form stable complexes with various transition metal ions that easily coordinate with nitrogen and oxygen atoms that provide versatile coordination abilities with the preparation of exciting mono- and polynuclear compounds, which provide possibilities for different applications [27,28,29,30]. Ganguly et al. [31] discussed the mode of binding and coordination of different multidentate reduced Schiff base ligands with metal ions such as Cu(II), Zn(II), and Ni(II) that offered the possibility of the construction of new supramolecular structures. Kitaura et al. [32] reported a novel three-dimensional (3D) microporous coordination framework obtained from Schiff base type ligands with CuII, NiII, and CoII ions, providing a new type of porous compound that could have applications in highly selective molecular recognition and heterogeneous catalysis. However, there are few reports on Schiff base coordination in the mid-far infrared field. Therefore, it is of great significance to thoroughly research the application of Schiff base materials in the field of stealth.

In this paper, we synthesized a novel polymer by taking advantage of the polycondensation reaction between polyetheramine and 2,6-pyridinedicarboxaldehyde. Furthermore, a series of polymer coatings were designed through the coordination of polymers and various transition metals, such as Ni^2+^, Cu^2+^, and Sm^3+^ ions. The comprehensive properties of the resulting infrared stealth coatings were characterized and the relationship between the coating structure and infrared emissivity was elaborated in detail.

## 2. Materials and Methods

### 2.1. Materials

All chemical reagents were purchased from Sigma-Aldrich (Shanghai, China) and solvents were purchased from Energy Chemical (Shanghai, China). They were used without further purification. All other starting materials were commercially available and used without any further purification.

### 2.2. Measurements

^1^H-NMR spectra were recorded using a Brucker (400 MHz) spectrometer (Bruker, Billerica, MA, USA) at 300 K using CDCl3 as a solvent. Infrared spectra were measured using a 6700 and a Nicolet IS50 spectrometer (Thermo Fisher, Waltham, MA, USA). The Ni, Cu, and Sm concentrations in polymers were determined by Optima2000DV Inductively Coupled Plasma (ICP) (PerkinElmer, Waltham, MA, USA). UV-Vis absorption spectra were recorded on a Lambda 750S UV-Vis spectrometer (PerkinElmer, Waltham, MA, USA). The chemical compositions of the polymers were characterized using ESCALAB 250Xi X-ray photoelectron spectroscopy (XPS) (Thermo Fisher, Waltham, MA, USA), and the XPS spectra were calibrated using the binding energy of C 1s (284.6 eV). The electrical conductivity of polymers was measured with the JG ST2742B electric powder resistivity tester (Jingge Electronic Co., Ltd., Suzhou, China) using the four-probe method. Mid-far infrared emissivity was measured using a TSS-5X Infrared Emissometer (Japan Sensor, Tokyo, Japan) at room temperature.

Thermogravimetric analysis (TGA) of the polymer coatings was performed on a Mettler TGA/SDTA851 thermogravimetric analysis instrument in a nitrogen atmosphere at a heating rate of 20 °C min^−1^ from 30 to 800 °C. The mechanical properties for polymer coatings were performed on a Hysitron TI 900 nanoindentation system equipped with a Berkovich diamond indenter at 500 nm depth. The impact resistance of polymer coatings was measured via a QCJ impact tester according to national standards GB/T 1732–2020 and the flexibility test was carried out by a QTX type paint film elasticity tester, in accordance with the national standard GB1731-93, using the tinplate substrates (12 cm × 5 cm × 0.28 mm) as the coating substrate. The adhesion force was evaluated using a grid-drawing tester according to International Standard ISO 2409-1992. The standard ranks the adhesive force in six grades, ranging from 0 to 5. Grade 0 represents the strongest adhesive force. Grade 5 represents the weakest adhesive force. Scanning electron microscopy (SEM) images were observed by using a SU8220 instrument (Hitachi, Tokyo, Japan) at 10 kV. Samples were sputter-coated with a layer of gold.

### 2.3. Synthesis of Polymers

The polymers containing Schiff base moieties were synthesized by solution polycondensation under a nitrogen atmosphere. A quantity of 10 mmol of polyetheramine (D230) dissolved in 10 mL of DMF was added to three 100 mL flasks. Then, a solution of 10 mmol of 2,6-pyridinedicarboxaldehyde dissolved in 20 mL of DMF was added dropwise and stirred for 10 h at 120 °C. When the reaction was completed, the polymer solution was cast on the glass plate and heated to remove the reaction medium. Then, it was washed with water and ethanol to remove unreacted monomers and dried under a vacuum at 80 °C. The polymer (D230-PyAL) was obtained after the removal of the solvent. The yield was up to 90%.

### 2.4. Preparation of the Polymer Coatings

Using D230-PyAL-Ni as an example: D230-PyAL, nickel acetate tetrahydrate, and DMF were added to a sample bottle. After low-temperature ultrasonic processing for 1 h, the solution was poured onto a substrate at 80 °C for 12 h. The solvent was slowly evaporated, and the coating was formed. D230-PyAL-Cu and D230-PyAL-Sm were prepared in the same way as above.

## 3. Results and Discussion

### 3.1. Synthesis of Polymers and Coordination Polymers

**Scheme 1:**
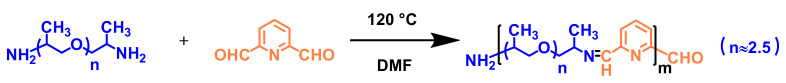
Synthesis of a Schiff base polymer.

**Figure 1:**
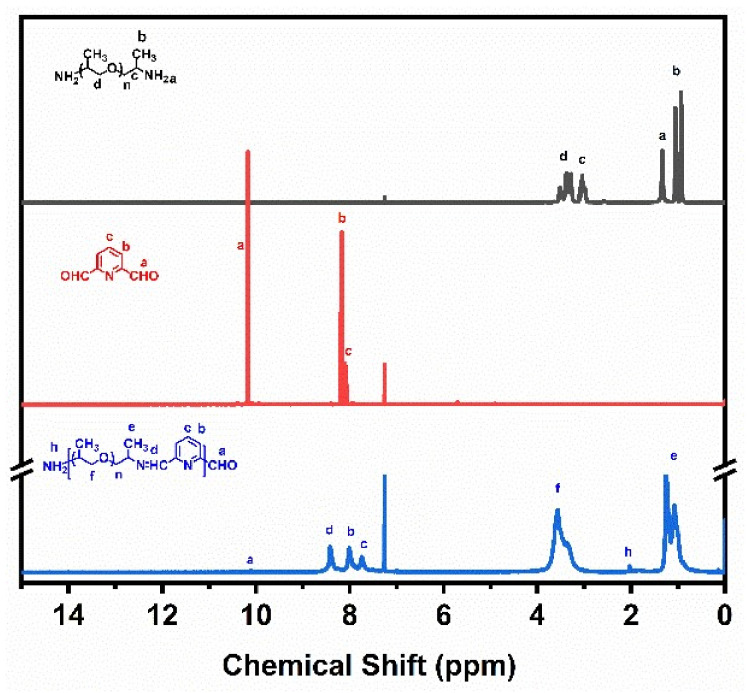
^1^H NMR spectra of monomers and Schiff base polymers in CDCl_3_.

The Schiff base polymer was obtained by condensation reaction between polyetheramine (D230) and 2,6-pyridinedicarboxaldehyde as shown in Scheme 1. ^1^H NMR spectra in Figure 1 showed the characteristic peaks of the amino group at 1.34 ppm and the aldehyde group at 10.17 ppm. After the condensation reaction in DMF for 12 h, the Schiff base polymer characteristic peaks of –NH_2_ and –CHO were attenuated, and at the same time, the –CH=N– peak appeared at 8.42 ppm. The results demonstrated the Schiff base polymer was successfully synthesized.

**Scheme 2:**
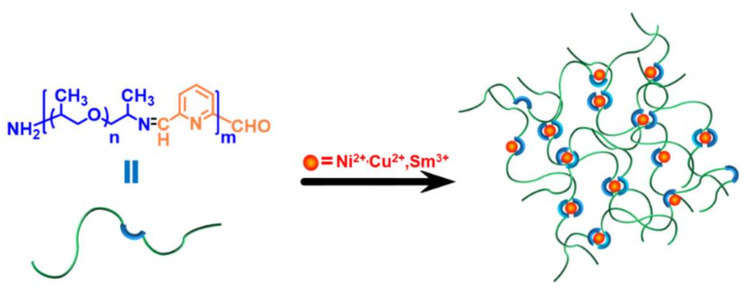
Schematic structures of Schiff base coordination polymers.

**Figure 2:**
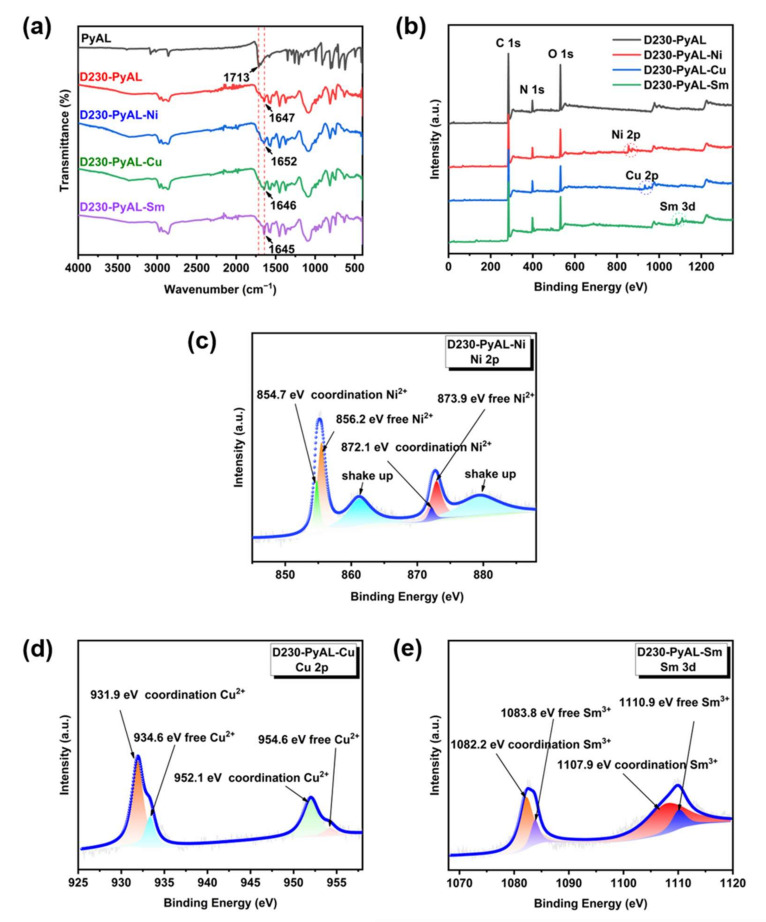
(**a**) FTIR and (**b**–**e**) XPS spectra of polymers and coordination polymers.

Schiff base polymers can coordinate with electron-deficient metal ions by donating electrons [33,34]. Thus, we synthesized its coordination polymers by introducing Ni^2+^, Cu^2+^, and Sm^3+^ ions (Scheme 2). The structures of three coordination polymers were investigated with FTIR spectra shown in Figure 2a. As could be seen, the disappearance of the aldehyde group (–C=O–) with a characteristic absorption peak of PyAL at 1713 cm^−1^ and the emergence of the imine bond (–C=N–) stretching vibration at 1647 cm^−1^ indicated the successful condensation reaction between D230 and PyAL monomers. After coordination with Ni^2+^, Cu^2+^, and Sm^3+^ ions, the absorption band of the imine bond slightly changed to 1652 cm^−1^,1646 cm^−1^, and 1645 cm^−1^, respectively. XPS spectra were used to further characterize the coordination structure in Figure 2b–e. The D230-PyAL polymers presented obvious characteristic peaks of C 1s, N 1s, and O 1s with relatively high peak intensities due to their high content of C, N, and O elements. The characteristic peaks of Ni 2p (eV), Cu 2p (eV), and Sm 3d (eV) apparently appeared in the XPS full spectra, respectively, which indicated that the metal ions have been successfully introduced into the polymer coatings. The metal element area of the coatings was fitted by peak separation and compared with the metal salt itself (Supporting information Figures S1–S3) shown in Figure 2c–e. It was found that there was a certain degree of metal coordination structure in the coating. The binding energies of the 2p3/2 and 2p1/2 Ni (II) peaks in Nickel(II) acetate tetrahydrate were 856.2 and 873.9 eV, respectively [35,36]. After coordination with the polymer, the binding energy decreased to 854.7 and 872.1 eV, respectively. After coordination, the binding energies of Cu 2p3/2 and Cu 2p1/2 in Copper(II) acetate decreased from 934.6 and 954.6 eV to 931.9 and 952.1 eV, respectively [37,38]. The binding energy peaks at 1083.8 and 1110.9 eV correspond to Sm 3d5/2 and Sm 3d3/2 in Samarium(III) acetate hydrate, respectively [39,40]. After coordination, Sm 3d5/2 peak and 3d3/2 peak, respectively, decreased to 1082.2 and 1107.9 eV. It was attributed to some electrons on the N atom entering the unoccupied orbital of the metal after coordination, resulting in an increase in the electron cloud density and a decrease in the corresponding binding energy [41]. Therefore, through the comparative analysis of the FTIR and XPS spectra, it was sufficient to prove that D230-PyAL, D230-PyAL-Ni, D230-PyAL-Cu, and D230-PyAL-Sm were successfully synthesized. The Ni^2+^, Cu^2+^, and Sm^3+^ ion content of the coordination polymers was determined by ICP testing. The coordination content of Ni^2+^ ions was at least 1.2%, and the coordination content of Cu^2+^ and Sm^3+^ ions was almost 3.8% and 3.6%, respectively.

### 3.2. UV-Vis Absorption Spectroscopic Analysis of Coordination Polymers

**Figure 3:**
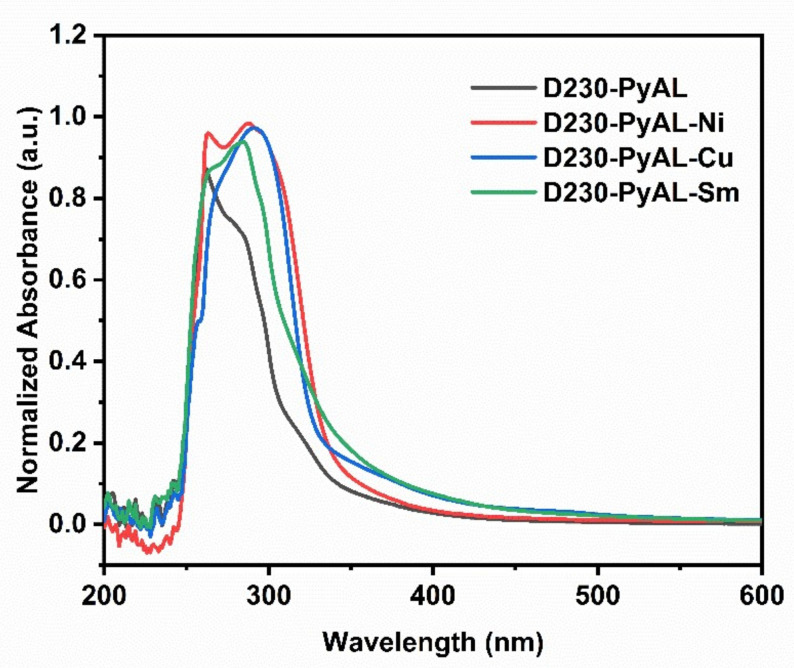
UV-Vis absorption spectra of polymers and coordination polymers.

The UV-Vis absorption spectra of D230-PyAL, D230-PyAL-Ni, D230-PyAL-Cu, and D230-PyAL-Sm are displayed in Figure 3. The intense absorption was observed at a wavelength of 263 nm, which could be assigned to the n–π* transition of imine bonds [34]. After coordination with Ni^2+^, Cu^2+^, or Sm^3+^, the peak intensity apparently decreased while a new absorption peak appeared at 287 nm, 291 nm, and 285 nm, respectively. This result indicated that the charge transfer and conjugation effect occurred between the imine group and metal ion [42,43]. The onset of UV-Vis absorption spectra determined the optical band gaps of D230-PyAL, D230-PyAL-Ni, D230-PyAL-Cu, and D230-PyAL-Sm [44,45]. The corresponding data is shown in Table 1. It was obvious from Table 1 that the onset absorptions of D230-PyAL, D230-PyAL-Ni, D230-PyAL-Cu, and D230-PyAL-Sm were 359, 374, 365, and 414 nm, corresponding to optical band gaps of 3.45, 3.32, 3.40, and 2.99 eV, respectively. This result showed that the introduction of metal ions into the polymer for coordination was beneficial in reducing the molecular band gap.

### 3.3. Electrical Conductivity of Polymers and Coordination Polymers

**Figure 4:**
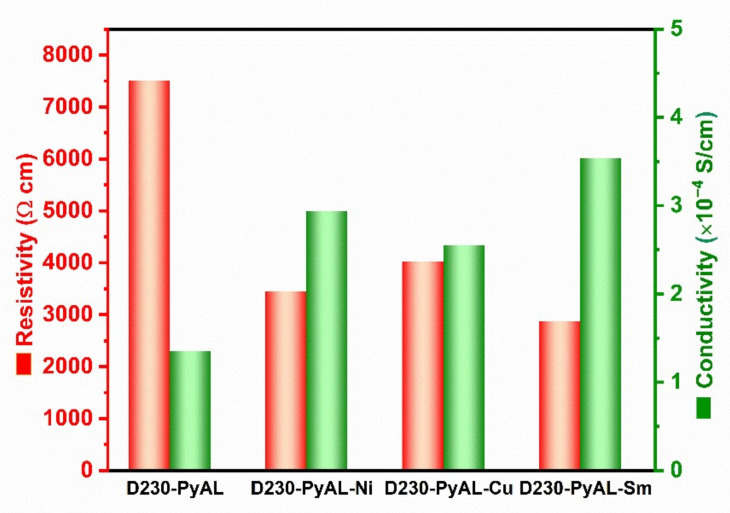
Resistivity and conductivity graphs for polymers and coordination polymers.

The electrical conductivity values of D230-PyAL, D230-PyAL-Ni, D230-PyAL-Cu, and D230-PyAL-Sm were measured using the four-point measurement technique. The obtained conductivity results are given in Figure 4 and Table 2. It was found that the electrical conductivity of D230-PyAL was 1.35 × 10^−4^ S/cm. After the coordination of metal ions, the resistance of D230-PyAL-Ni, D230-PyAL-Cu, and D230-PyAL-Sm decreased from 7508 Ω cm (D230-PyAL) to 3443 Ω cm, 4029 Ω cm, and 2873 Ω cm, respectively. Meanwhile, the electrical conductivity has been significantly increased. Its values were 2.94 × 10^−4^ S/cm, 2.55 × 10^−4^ S/cm, and 3.54 × 10^−4^ S/cm, respectively. Due to the coordination reaction between the imine bond and the metal ion, charge transfer occurred, resulting in the generation of charged points on the polymer molecular chain and an improvement in the conductivity of polymers [42,46]. Among them, it could be seen that the maximum conductivity value was obtained when Sm^3+^ was introduced, and the conductivity of D230-PyAL-Sm increased by 162% compared with D230-PyAL. This result was consistent with that of UV-Vis absorption, proving that a larger ionic radius of the cation may lead to higher conductivity [42].

### 3.4. Infrared Emissivity

The infrared emissivity of D230-PyAL, D230-PyAL-Ni, D230-PyAL-Cu, and D230-PyAL-Sm coatings in the 2–22 μm band region is shown in Table 3. The infrared emissivity of D230-PyAL was 0.70. After coordination with Ni^2+^, Cu^2+^, or Sm^3+^, the emissivity decreased with the difference in the metal ions. The minimum emissivity of the D230-PyAL-Sm coating was 0.58, which aligned with the experiment results of UV-Vis absorption and electrical conductivity. This result indicated that reducing the molecular bandgap and increasing the conductivity of the polymer through the coordination reaction was beneficial to reducing the infrared emissivity of the polymer coatings.

**Figure 5:**
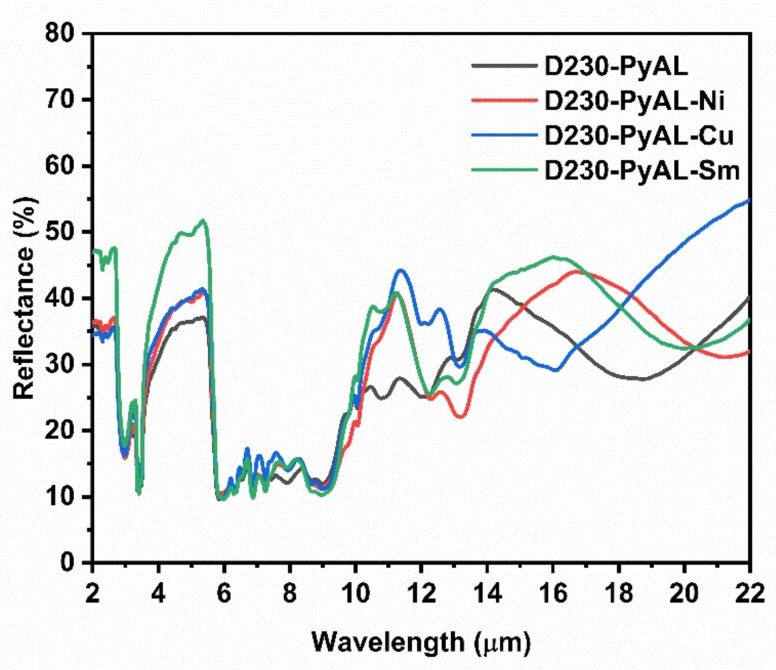
Reflectance curves of polymers and coordination polymer coatings.

At the same time, the emissivity of the coating was indirectly studied through reflection, as shown in Figure 5. According to Kirchhoff’s law, the emissivity (ε) is equal to absorptivity (a) under the condition of thermal equilibrium. Therefore, the decrease in infrared absorption leads to lower emissivity. From Figure 5, the average reflectivity of D230-PyAL, D230-PyAL-Ni, and the D230-PyAL-Cu coatings was about 30%, while the D230-PyAL-Sm coating was about 40% at 2–22 μm, which indicated that the D230-PyAL-Sm coating had lower emissivity, which was consistent with the infrared emissivity test results.

### 3.5. Thermal Properties

**Figure 6:**
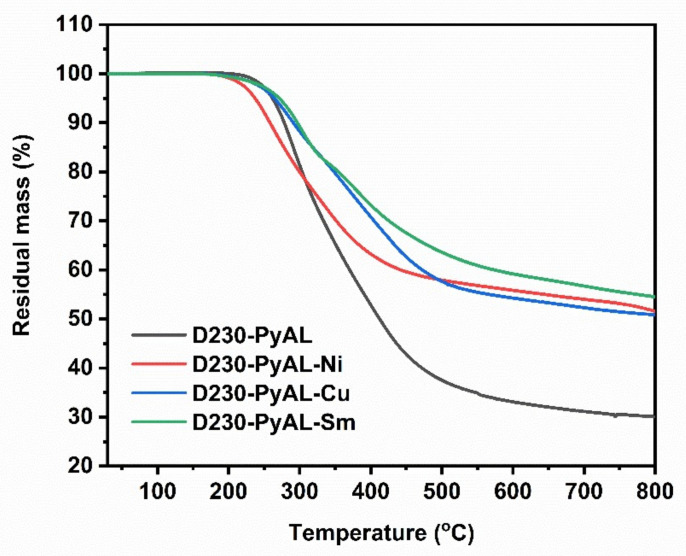
TGA images of polymers and coordination polymers.

The thermal properties of D230-PyAL, D230-PyAL-Ni, D230-PyAL-Cu, and D230-PyAL-Sm coatings were evaluated by thermogravimetric analysis (TGA) under an N_2_ atmosphere at a heating rate of 20 °C min^−1^ as shown in Figure 6 and Table 4. For D230-PyAL, the temperature for 5% weight loss (Td5%) was determined to be 262 °C. As the temperature was increased above T_d5%_, the weight loss increased abruptly, indicating the decomposition of the polymer backbone. The 5% decomposition temperatures of D230-PyAL-Ni, D230-PyAL-Cu, and D230-PyAL-Sm after coordination with Ni^2+^, Cu^2+^, or Sm^3+^ were 237, 264, and 271 °C, respectively. The thermal properties of D230-PyAL-Cu and D230-PyAL-Sm coatings were improved after the introduction of Cu^2+^ and Sm^3+^ ion coordination, respectively. However, it could be seen that the 5% weight loss temperature of the D230-PyAL-Ni was lower than that of the D230-PyAL. On the one hand, the coordination content of Ni^2+^ was less. On the other hand, metal ions could catalyze the degradation of the polymer [47]. The char yields of D230-PyAL-Ni, D230-PyAL-Cu, and D230-PyAL-Sm, calculated as the percentage of solid residue after heating from 30 to 800 °C in flowing nitrogen, were significantly higher than that of D230-PyAL (30%), at 52%, 51%, and 54%, respectively.

### 3.6. Mechanical Properties

**Figure 7:**
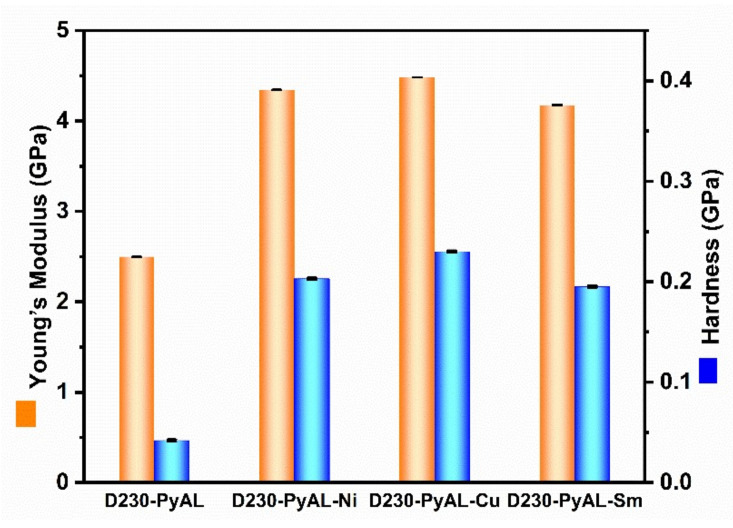
Young’s modulus (E) and hardness (H) are calculated from the nanoindentation.

Nanoindentation was used to measure nanomechanical properties of polymer coatings by analyzing Young’s modulus (E) and hardness (H) at a constant depth (500 nm). The average obtained nanoindentation test results are shown in Figure 7 and Table 5. The coordination polymer coating’s Young’s modulus and hardness were much higher than that of the D230-PyAL coating (2.498 ± 0.001 GPa and 0.042 ± 0.001 GPa). The E values of D230-PyAL-Ni, D230-PyAL-Cu, and D230-PyAL-Sm coating were 73.85%, 79.46%, and 67.13% higher than those of D230-PyAL coating, and the hardness was 383%, 447%, and 364% higher than that of D230-PyAL coating. Furthermore, we studied the plasticity index (the ratio of hardness to elastic modulus) to determine the elastic behavior of the contact surfaces; the higher the H/E ratio, the better the wear resistance. The H/E ratio for D230-PyAL was 0.016. The introduction of coordination ions improves the wear resistance of the polymer coating surface. It might be due to the introduction of metal ions to enhance the degree of cross-linking within polymer coatings [48]. The highest H/E ratio was observed for the D230-PyAL-Cu coating (0.051), compared to 0.047 for both the D230-PyAL-Ni and D230-PyAL-Sm coatings. It might be due to the strongest coordination ability of the D230-PyAL with copper ions.

To further investigate the mechanical properties of coatings, the flexibility, impact resistance, and adhesion of the coatings were evaluated as shown in Table 6. There is no significant crack or peeling of the coating of D230-PyAL and D230-PyAL-Sm when tested under the condition of bending on a 1 mm diameter shaft rod and a 50 cm heavy hammer impact, thus proving the excellent flexibility and impact resistance of these two coatings. The D230-PyAL-Ni coating was bent on a 1 mm diameter shaft rod with slight cracking but resisted the 50 cm impact test. However, the surface of the D230-PyAL-Cu coating had an obvious annular cracking and peeling phenomenon after bending on a 1 mm diameter shaft rod and impacting on a 50 cm heavy hammer. This might be because the high content of Cu^2+^ ions increased the coordination of the polymer, further increasing the rigidity of the polymer coating. The adhesion test of the coatings showed that D230-PyAL was grade 0. After the introduction of metal ion coordination, the adhesion level of the coating had dropped to grade 1. According to the performance evaluation standard of the coating system (ISO12944), the coating adhesion reaches grade 1 and meets the usage requirements.

### 3.7. SEM Micrographs of Coatings

**Figure 8:**
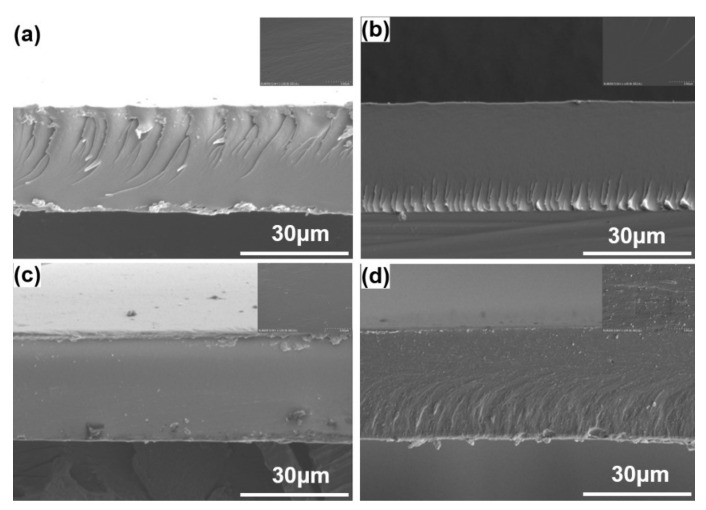
SEM images for section structures of (**a**) D230-PyAL, (**b**) D230-PyAL-Ni, (**c**) D230-PyAL-Cu, and (**d**) D230-PyAL-Sm coatings.

The section morphologies of polymer coatings make a difference in the mechanical properties, and the results are shown in Figure 8. The coating thickness was about 30 μm. As observed, the section morphologies of D230-PyAL, D230-PyAL-Ni, D230-PyAL-Cu, and D230-PyAL-Sm coatings were very homogeneous on a micro scale and no microphase separation or molecular aggregation appeared.

## 4. Conclusions

In this study, we prepared the polymers containing Schiff base moieties using polyetheramine and 2,6-pyridinedicarboxaldehyde by solution polycondensation and coordinated with Ni^2+^, Cu^2+^, and Sm^3+^ ions to prepare organic coatings. The corresponding structural characterizations were discussed by ^1^H-NMR, FTIR, XPS, and UV-Vis. The results showed polymers containing Schiff base moieties and coordination polymers were successfully synthesized. Moreover, the relationship between the molecular structure band gap and the conductivity and infrared emissivity of a polymer coating has been discussed in detail. UV-Vis and electrical conductivity measurements showed coordination polymers had lower molecular band gaps and higher electrical conductivity relative to uncoordinated polymers. The infrared emissivity of polymer coatings before and after coordination varied from 0.7 to 0.58 at wavelengths of 2–22 μm, indicating the improvement in the conductivity of polymers could contribute to the decrease in infrared emissivity. Furthermore, the thermal and mechanical properties were analyzed by TGA and nanoindentation tests. Additionally, the results showed that polymer coatings, coordinated with metal ions, had better thermal and mechanical properties than uncoordinated polymer coatings. Further research on the flexibility and impact resistance of the coating demonstrated that appropriate coordination ions could endow the coating with optimal flexibility and impact resistance. In summary, the polymer coating had the lowest energy band gap and the best electrical conductivity when it coordinated with Sm^3+^ ions. Meanwhile, the coating coordinated with Sm^3+^ ions had the lowest infrared emissivity in the 2–22 μm mid-far infrared waveband range, reaching 0.58, indicating that polymers containing Schiff base moieties and their complexes have a high potential for use as infrared stealth materials in military applications.

## Figures and Tables

**Table 1:** UV-Vis data of polymers and coordination polymers.

Polymer Name	λ_onset_	E_g_
	(nm)	(eV)
D230-PyAL	359	3.45
D230-PyAL-Ni	374	3.32
D230-PyAL-Cu	365	3.40
D230-PyAL-Sm	414	2.99

**Table 2:** Resistivity and conductivity values for polymers and coordination polymers.

Polymer Name	Resistivity	Conductivity
	(Ω cm)	(×10^−4^ S/cm)
D230-PyAL	7508	1.35
D230-PyAL-Ni	3443	2.94
D230-PyAL-Cu	4029	2.55
D230-PyAL-Sm	2873	3.54

**Table 3:** Infrared emissivity values of polymers and coordination polymer coatings.

Polymer Name	ε_2–22μm_
D230-PyAL	0.70
D230-PyAL-Ni	0.66
D230-PyAL-Cu	0.68
D230-PyAL-Sm	0.58

**Table 4:** TGA results for polymers and coordination polymers.

Polymer Name	T_d5%_	T_d10%_	Char Yield
D230-PyAL	262	278	30
D230-PyAL-Ni	237	258	52
D230-PyAL-Cu	264	291	51
D230-PyAL-Sm	271	297	55

**Table 5:** The results obtained from the nanoindentation test.

Polymer Name	E	H	H/E
	(GPa)	(GPa)	
D230-PyAL	2.498 ± 0.001	0.042 ± 0.001	0.016
D230-PyAL-Ni	4.343 ± 0.002	0.203 ± 0.001	0.047
D230-PyAL-Cu	4.483 ± 0.001	0.230 ± 0.001	0.051
D230-PyAL-Sm	4.175 ± 0.003	0.195 ± 0.001	0.047

**Table 6:** The results for flexibility, impact resistance, and adhesive force of D230-PyAL, D230-PyAL-Ni, D230-PyAL-Cu, and D230-PyAL-Sm coatings.

Polymer Name	Impact Resistance ^a^	Flexibility ^b^	Adhesive Force
	(cm)	(mm)	(Grade)
D230-PyAL	50	0.5	0
D230-PyAL-Ni	50	1	1
D230-PyAL-Cu	10	2.5	1
D230-PyAL-Sm	50	0.5	1

^a^ drop hammer height from sample; ^b^ shaft rod surface radius.

## Data Availability

The data presented in this study are available on request from the corresponding author.

## Supplementary Materials

The following supporting information can be downloaded at: https://www.mdpi.com/article/10.3390/polym14214563/s1, Figure S1: XPS spectrum of Nickel(II) acetate tetrahydrate; Figure S2: XPS spectrum of Copper(II) acetate; Figure S3: XPS spectrum of Samarium(III) acetate hydrate.
