# Supplementary material for: Synthesis and Characterization of Schiff Base Polymers via Metal Coordination and Its Application in Infrared Stealth Coating
Source: Polymers (Basel). 2022 Oct 27;14(21):4563. doi: 10.3390/polym14214563 (PMC9659303; doi:10.3390/polym14214563)
Supplement: Supplementary file 1 [file polymers-14-04563-s001.zip › polymers-1955767-supplementary.pdf]

Supplementary Materials

Synthesis and Characterization of Schiff Base Polymers via Metal Coordination and Its Application in Infrared Stealth Coating

Xiangyu Li, Lishuai Zong, Weijie Li, Yibo Wang, Jinyan Wang * and Xigao Jian

State Key Laboratory of Fine Chemicals, Liaoning High Performance Resin Engineering Research Center, Department of Polymer Science & Engineering, Dalian University of Technology, Dalian 116024, China
* Correspondence: wangjinyan@dlut.edu.cn

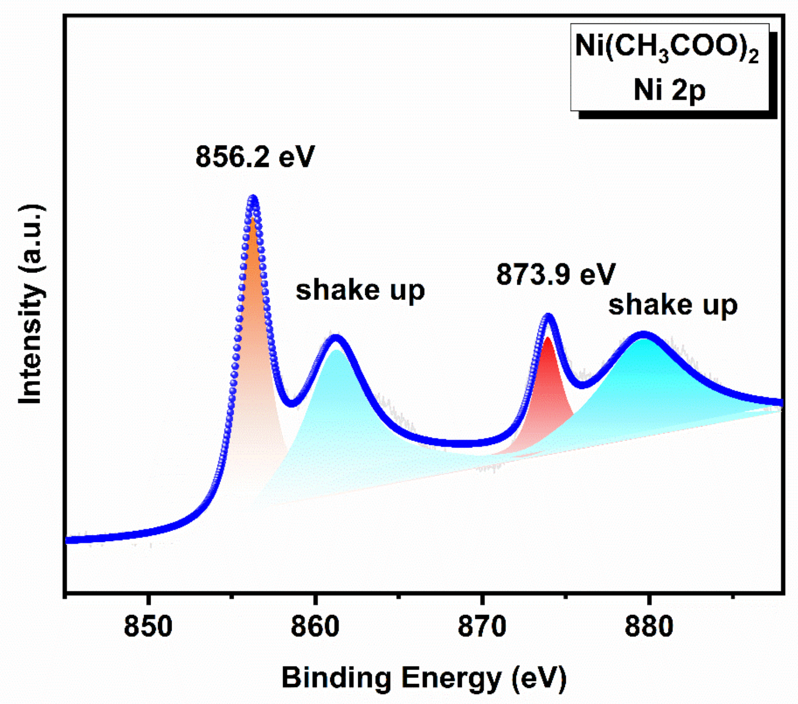

Figure S1. XPS spectrum of Nickel(II) acetate tetrahydrate.

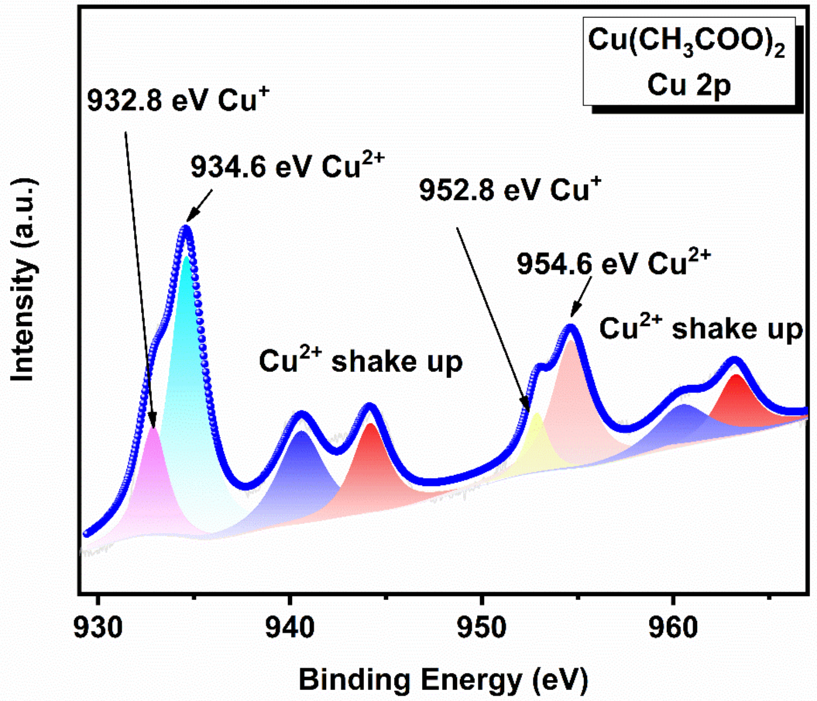

Figure S2. XPS spectrum of Copper(II) acetate.

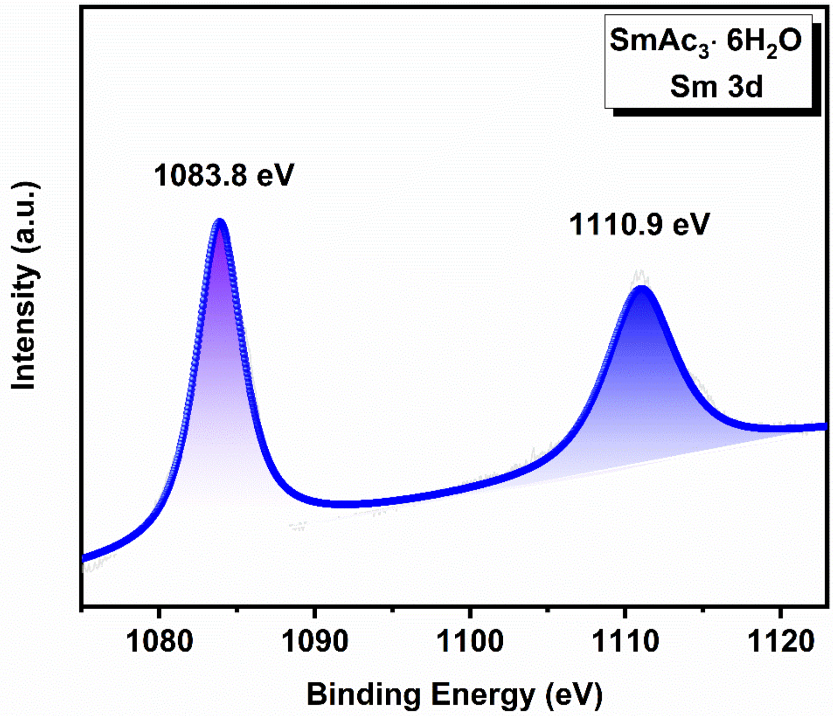

Figure S3. XPS spectrum of Samarium(III) acetate hydrate.
